# Evaluation of the Cytotoxicity of α-Cyclodextrin Derivatives on the Caco-2 Cell Line and Human Erythrocytes

**DOI:** 10.3390/molecules201119694

**Published:** 2015-11-11

**Authors:** Eszter Róka, Zoltán Ujhelyi, Mária Deli, Alexandra Bocsik, Éva Fenyvesi, Lajos Szente, Ferenc Fenyvesi, Miklós Vecsernyés, Judit Váradi, Pálma Fehér, Rudolf Gesztelyi, Caroline Félix, Florent Perret, Ildikó Katalin Bácskay

**Affiliations:** 1 Department of Pharmaceutical Technology, Faculty of Pharmacy, University of Debrecen, Nagyerdei körút 98, Debrecen H-4032, Hungary; roka.eszter@pharm.unideb.hu (E.R.); ujhelyi.zoltan@pharm.unideb.hu (Z.U.); fenyvesi.ferenc@pharm.unideb.hu (F.F.); vecsernyes.miklos@pharm.unideb.hu (M.V.); varadi.judit@pharm.unideb.hu (J.V.); feher.palma@pharm.unideb.hu (P.F.); 2 University Lyon 1, ICBMS, Equipe CSAp, 43 Boulevard du 11 Novembre 1918, Villeurbanne F-69622, France; caroline.felix@univ-lyon1.fr (C.F.); florent-perret@univ-lyon1.fr (F.P.); 3 Department of Biophysics, Biological Research Centre, Hungarian Academy of Sciences, Temesvári körút 62, Szeged H-6726, Hungary; deli.maria@brc.mta.hu (M.D.); bocsik@brc.hu (A.B.); 4 Cyclolab Ltd., Illatos út 7, Budapest H-1097, Hungary; fenyvesi.e@cyclolab.hu (É.F.); szente@cyclolab.hu (L.S.); 5 Department of Pharmacology and Pharmacodynamics, Faculty of Pharmacy, University of Debrecen, Nagyerdei körút 98, Debrecen H-4032, Hungary; gesztelyi.rudolf@pharm.unideb.hu

**Keywords:** α-CD derivatives, cytotoxicity, Caco-2 cell line, hemolysis, RT-CES

## Abstract

Cyclodextrins, even the 6-membered α-cyclodextrin, are approved in the various pharmacopoeias as pharmaceutical excipients for solubilizing and stabilizing drugs as well as for controlling drug release. Recently α-cyclodextrin has also been marketed as health food with beneficial effects on blood lipid profiles. However, the concentration of α-cyclodextrin used may be very high in these cases, and its toxic attributes have to be seriously considered. The objective of this study was to investigate the cytotoxicity of various, differently substituted α-cyclodextrin derivatives and determine relationship between the structures and cytotoxicity. Three different methods were used, viability tests (MTT assay and Real Time Cell Electronic Sensing on Caco-2 cells) as well as hemolysis test on human red blood cells. The effect of α-cyclodextrin derivatives resulted in concentration-dependent cytotoxicity, so the IC50 values have been determined. Based on our evaluation, the Real Time Cell Electronic Sensing method is the most accurate for describing the time and concentration dependency of the observed toxic effects. Regarding the cytotoxicity on Caco-2 cells, phosphatidylcholine extraction may play a main role in the mechanism. Our results should provide help in selecting those α-cyclodextrin derivatives which have the potential of being used safely in medical formulations.

## 1. Introduction

Cyclodextrins (CDs) are widely used excipients and still in the focus of drug development [1]. They are used as solubilizing factors, protective agents for light-sensitive drugs, and as a part of sustained release or drug delivery systems [2]. In some cases their stabilizing effect against hydrolysis, oxidation, and microbial decomposition is utilized. They can also reduce the bitter taste and unpleasant odor of the active compounds they include. There are several products on the market (tablets, eye drops, transdermal patches, inhalers, *etc.*) which contain various CDs already approved as pharmaceutical ingredients [3,4]. Most of these products contain β-CD or its hydroxypropyl, and sulfobutyl derivatives, and only a few of the marketed formulations are produced with α-CD. Nowadays, the favorable effect of orally administered α-cyclodextrins on blood lipids and weight loss in healthy humans has been recognized [5]. Although α-CD like β-CD is practically not absorbed from the gastrointestinal tract explaining the low oral toxicity, it might damage the cells of the intestine, especially at the high applied concentrations used in these applications. 

In the case of β-CD and its derivatives the toxic effects on living organisms are attributed to the affinity to cholesterol [6]. *In vitro* studies have shown that this phenomenon is behind the hemolytic effect of β-CDs, too [7]. The hemolytic effect of the non-cholesterol interacting α-CD was explained by its capability of forming inclusion complexes with other membrane lipid constituents such as phospholipids [8]. β-CDs have the most significant hemolytic activity; in the case of α- and γ-derivatives it is less considerable while δ-CD is not hemolytic at all [9]. The substituents on the CD derivatives may modify (increase or decrease) these effects depending on the lipid solubilizing properties [9,10,11,12]. For instance, the hemolytic activity of α-CD derivatives on rabbit’s red blood cells was enhanced by methylation and reduced by hydroxypropylation to follow the order of dimethyl α-CD > α-CD > hydroxypropyl α-CD, which correlates with extraction of phospholipids including sphingomyelin and of proteins from the membrane [13].

The hemolytic activity has been thoroughly investigated, but only a few studies on CDs’ cytotoxicity on other cell cultures have been reported. In pulmonary Calu-3 cells the methylated β-CD was the most toxic, while the hydroxypropylated α-CD and β-CD, as well as the native γ-CD proved to be safe for pulmonary drug delivery [14]. Evaluating the cytotoxicity of natural CDs and hydroxypropylated derivatives on P388 murine leukaemic cells a similar order of cytotoxicity was observed as in erythrocytes, in spite of the biological differences between the membranes of these cells [8]. Toxic effects of several β-CD derivatives have been studied on Caco-2 cells, and like red blood cells, a strong correlation was found between the cholesterol solubilizing effect and the cytotoxicity [11,12]. The cytotoxic attributes depend not only on the properties of the CDs (number of glucopyranose units, the chemical nature of the substituent, degree and pattern of substitution, the HLB value, the applied concentration), but also on the duration of exposition, the presence of serum components and density of the cells [15].

Not only pure CDs and their derivatives have been thoroughly investigated, but there are several studies on the cytotoxicity of CD complexes, too. In early works of Uekama’s group it was proved that complexation decreases the hemolytic activity of the drug encapsulated via decreasing the concentration of the free drug able to interact with the cell membrane [16].

Some recent examples: sevoflurane-sulfobutylether-β-CDs showed no toxic effect on brain microvascular endothelial cells [17], midazolam-trimethyl-β-CD complex was not toxic on cEND cells [18]. On the other hand, there are examples when CDs do not influence the toxic effects of drugs or show even enhanced effect on cancer cell lines. For instance, curcumin complexed by β-CD was effective in inhibiting the cell proliferation in lung (A-459) and colon (SW-620) cancer cell lines determined via MTT assay and enhanced *in vitro* toxicity (anticancer activity) of resveratrol was observed when complexed by sulfobutyl ether β-CD on a human breast cancer cell line (MCF-7) [19,20].

Nanoparticles containing CDs can go through the biological barriers and can be used as targeted drug delivery systems [1]. For instance, β-CDs-poly(β-amino ester) formed non-toxic nanoparticles, which can transport drugs across blood-brain barrier to treat chronic diseases in the brain [21]. Although, β-CDs are the most frequently used representatives of CDs in the pharmaceutical and food industries, which implicitly results in a broad range toxicological studies of numerous derivatives, amphiphilic α-CDs can also form self-assembled nanoparticles [22]. Some fluorinated amphiphilic α-CD successfully improved the stability of lipophilic antitumor drugs and presented high *in vivo* tolerance [23].

In addition to the human blood cells we selected Caco-2 cells for our studies. Caco-2 cells are considered a reliable model of orally administered pharmacons [21]. These cells are of colonic origin, they express similar drug transporters to the human intestine [24,25] and exhibit a well differentiated brush border on the apical surface and tight junctions [26]. A strong correlation was observed between *in vivo* human absorption and *in vitro* P_app_ (apparent permeability coefficient) for a variety of compounds encompassing transcellular, paracellular and carrier-mediated mechanisms [27]. Novel *in vitro* Caco-2 hepatocyte hybrid system for predicting *in vivo* oral bioavailability was developed resulting in more reliable and stronger correlation between *in vitro* and *in vivo* data [28,29,30].

As there are only sporadic data on the cytotoxicity of α-CD derivatives we started a systematic study aiming at evaluating the cytotoxicity of various α-CD derivatives on human erythrocytes and Caco-2 cell monolayer and exploring the relationship between the structures and cytotoxicity. Three different methods were used: viability tests (MTT assay and hemolysis test), and real time cell electronic sensing assay (RT-CES). Viability tests are useful for obtaining information about the concentration-dependence of the mechanism, and RT-CES is an adequate method to understand the time-dependent mechanism of cell degradation.

## 2. Results and Discussion

### 2.1. Results

#### 2.1.1. Hemolytic Effect of Different α-CD Derivatives

Native α-CD showed relatively high hemolytic effect as its HC50 value is 16 mM. TRIMEA was much more hemolytic followed by the succinyl derivative. RAMEA caused hemolysis at similar concentration to the non-substituted native α-CD. The order of hemolytic effect in case of methylation is TRIMEA >> RAMEA > native α-CD. Among the non-ionic derivatives HPACD and AcACD were not hemolytic up to 100 mM concentration.

**Figure 1:**
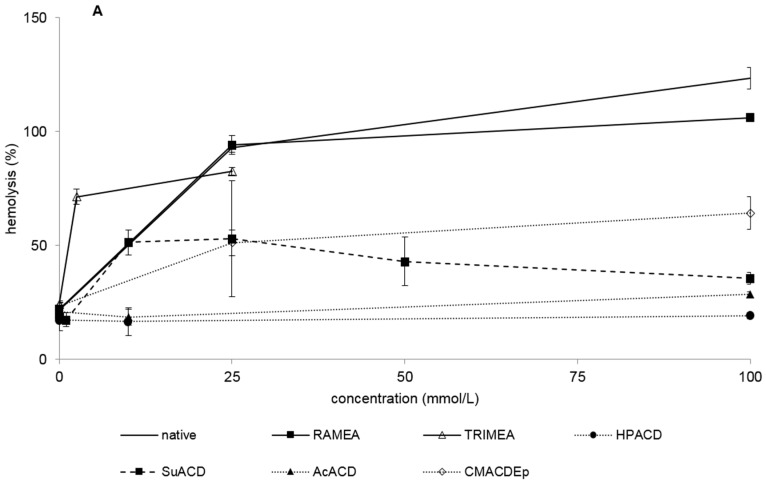

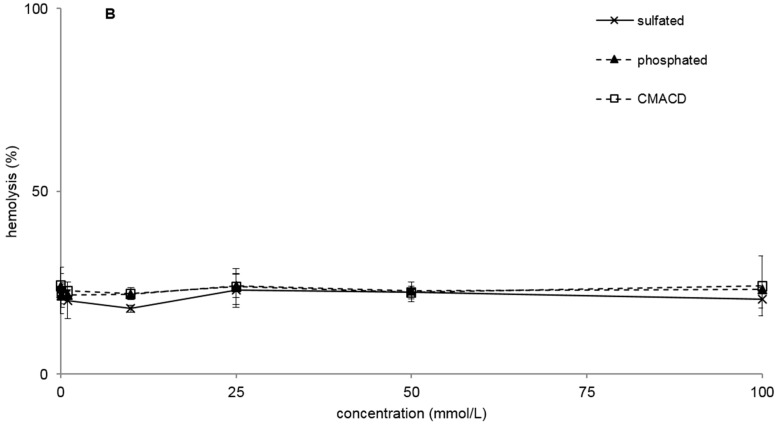
Hemolytic effect of non-ionic (**A**) and ionic (**B**) α-CD derivatives on human red blood cells. Hemolysis was expressed as the percentage of untreated control in the function of α-CD derivatives concentration. Negative control: PBS. Positive control: Purified water. Values presented are means ± SD. All data were obtained from three to five independent biological replicates and in the same experiments, four parallel concentrations were measured.

In case of the ionic derivatives (phosphated, sulfated and carboxymethylated), high HC50 values (>100 mM) were determined, which indicates that there is no severe hemolytic effect under these circumstances (Figure 1A,B and Table 1).

**Table 1:** Comparison of α-CD derivatives cytotoxicity by different methods (MTT, RT-CES, hemolysis). Values are in mM, and expressed as mean ± SD. All data were obtained from three to five independent biological replicates and in the same experiments four parallel concentrations were measured.

α-Cyclodextrin Derivative	IC50 (MTT)	IC50 (RT-CES)	HC50
native	46.1 ± 9.2	>25	16.0 ± 0.02
RAMEA	78.6 ± 15.8	>25	15.5 ± 0.01
TRIMEA	1.8 ± 0.8	>1	1.9 ± 0.01
HPACD	>100	>100	>100
sulfated	>100	>10	>100
phosphated	7.8 ± 8.6	>10	>100
CMACD	>100	>25	>100
SuACD	19.0 ± 8.8	>1	9.6 ± 0.03
AcACD	>100	>100	>100
CMACDEp	58.4 ± 0.4	>10	24.5 ± 0.01

#### 2.1.2. Effect of α-CD Derivatives on Cell Viability

**Figure 2:**
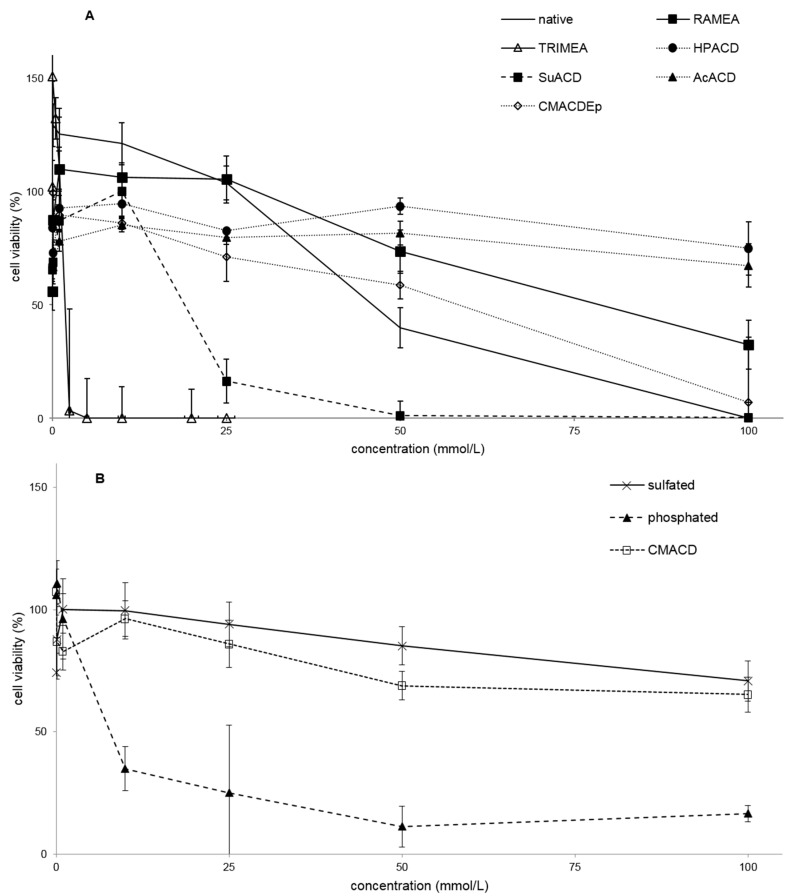
Effect of non-ionic (**A**) and ionic (**B**) α-CD derivatives on Caco-2 cell viability, determined by MTT-test. Cell viability was expressed as the percentage of untreated control in the function of α-CD derivatives concentration. Phosphate buffered saline (PBS) served as negative control and Triton X 100 (10% *w*/*v*) as positive control. Values presented are means ± SD. All data were obtained from three to five independent biological replicates and in the same experiments four parallel concentrations were measured.

The MTT assay showed similar toxic effects of α-CDs as the hemolysis test (Figure 2A,B, and Table 1), although some derivatives show remarkable differences (e.g., phosphated Na-salt). This phosphated sodium salt did not result in hemolysis but in MTT assay it was very toxic presenting IC50 concentration of 7.8 mM. TRIMEA was the most toxic in both tests, while RAMEA was less toxic than the native α-CD. In the case of the two methylated derivatives the following order was recognized in toxicity: TRIMEA > native α-CD > RAMEA. On the other hand, HPACD, AcACD, sulfated, CMACD derivatives did not show toxic effect up to 100 mM just like they had no hemolytic effect either.

**Figure 3:**
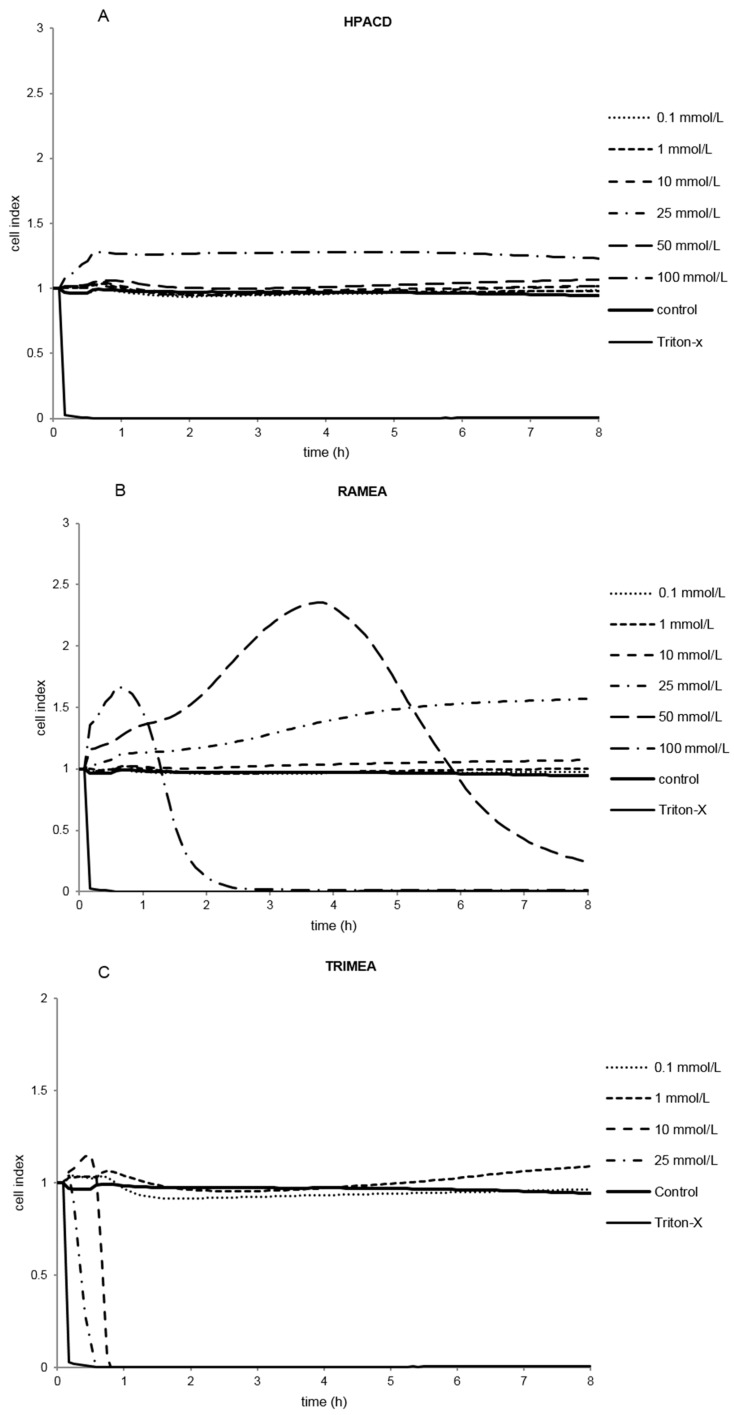

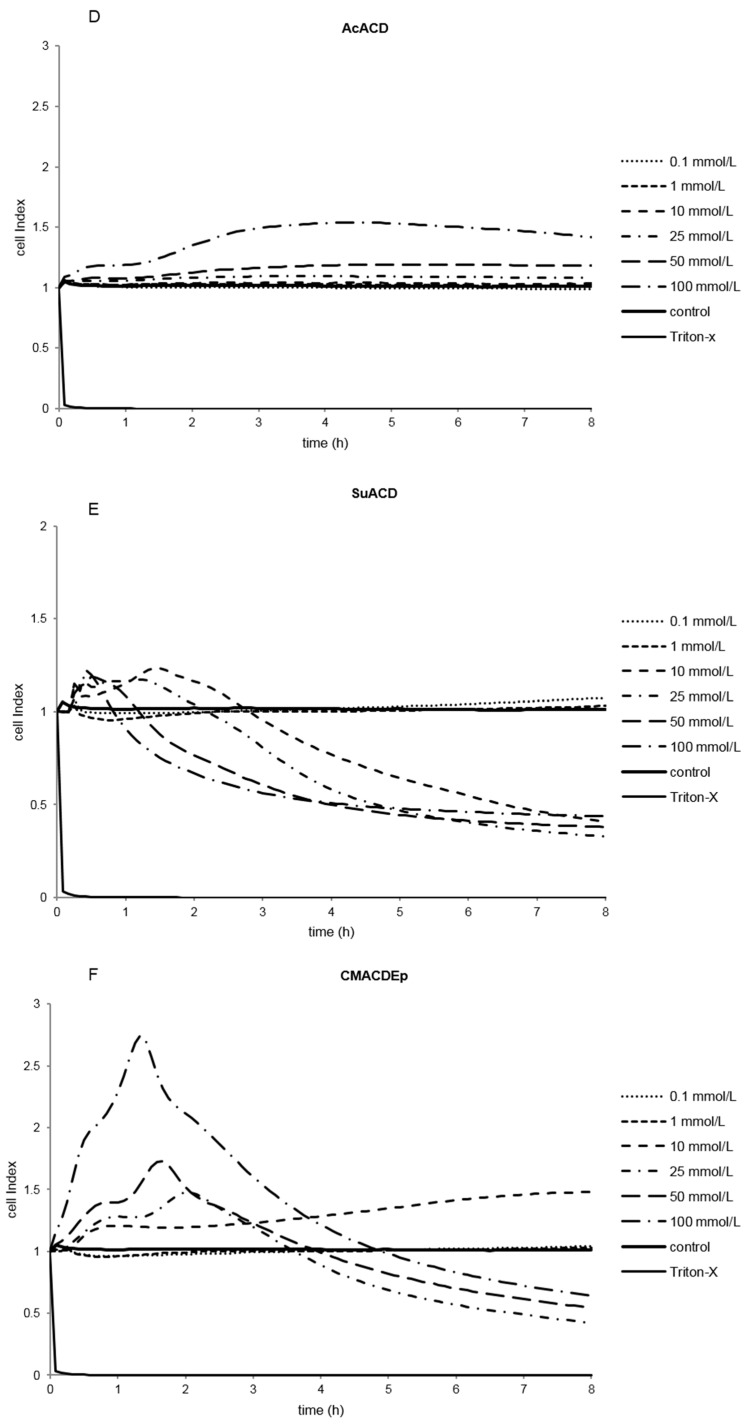

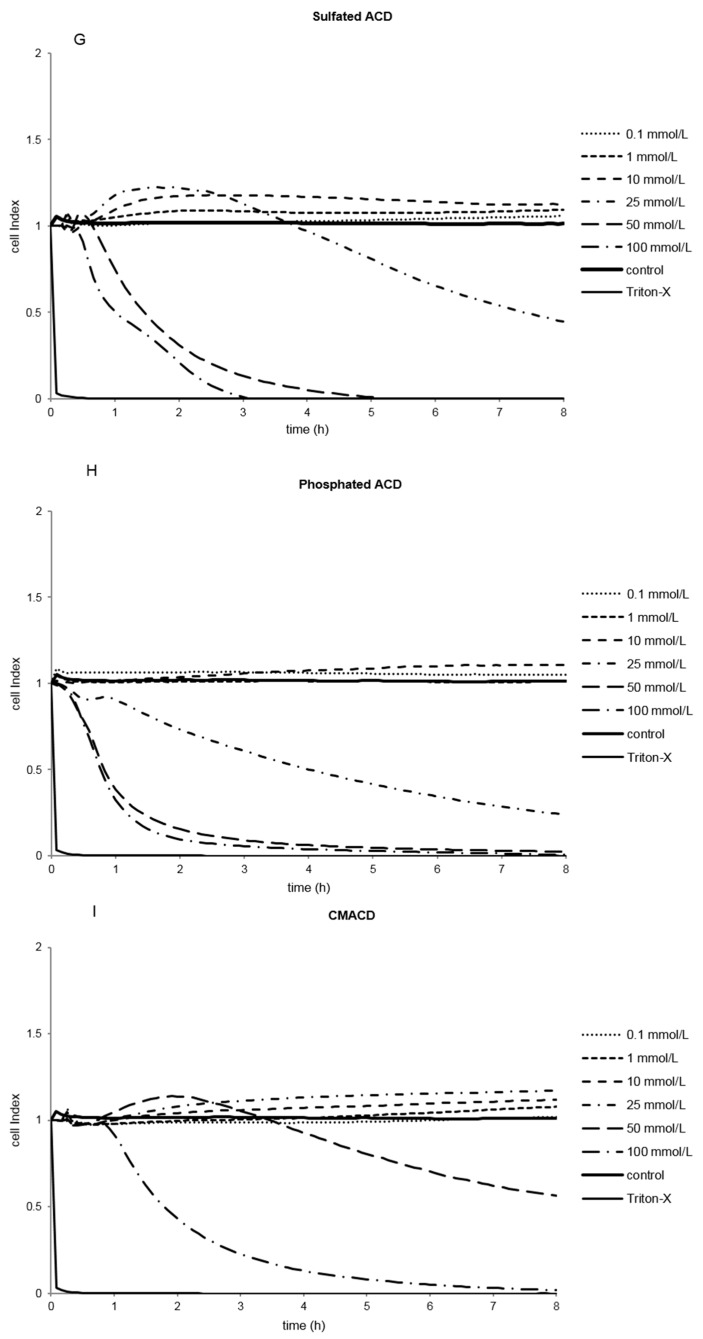
Effect of non-ionic (**A**–**F**) and ionic (**G**–**I**) α-CD derivatives on Caco-2 cell viability, determined by Real Time Cell Electronic Sensing. Changes in cell index indicating viability of Caco-2 cells up to 8 h treatment with different α-CD derivatives. Data are presented as mean ± S.D: *n* = 3 parallel samples. Positive control: 1% Triton X-100 detergent. Negative control: PBS.

Several derivatives showed measurable IC50 values with the RT-CES method (Figure 3). Only HPACD and the AcACD derivative remained non-toxic up to 100 mM. For example, CMACD and sulfated ACD had no defined IC50 value by the MTT assay up to 100 mM after 30 min of treatment, but they were toxic when the exposition time was prolonged. In case of CMACDEp IC50 value could be determined with the previous method as well, but it decreased with the same time prolongation.

#### 2.1.3. Indirect Verification of α-CD Cytotoxicity on Caco-2 Cells

**Figure 4:**
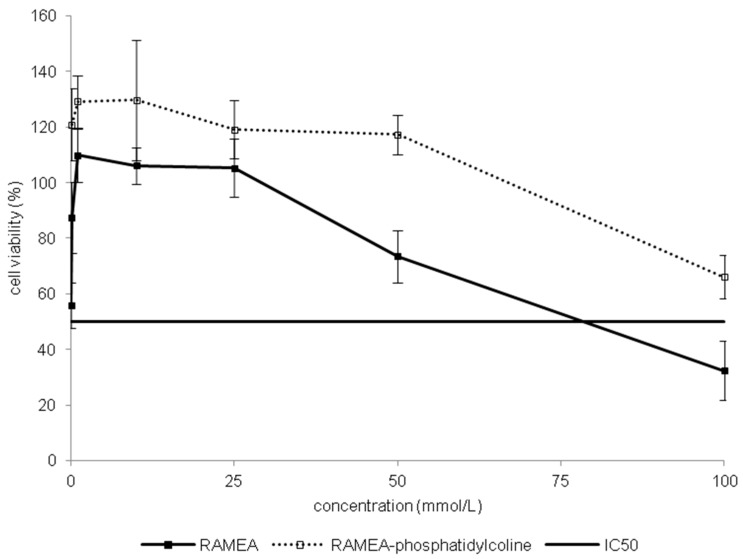
Comparison of toxicity on Caco-2 cells of RAMEA and RAMEA–phosphatidylcoline complex. Cell viability was expressed as the percentage of untreated control in the function of α-CD derivatives concentration. Values presented are means ± SD. All data were obtained from three to five independent biological replicates and in the same experiments four parallel concentrations were measured.

Toxic effect of RAMEA and pre-formed inclusion complex of phosphatidylcholine:RAMEA was compared by MTT assay on Caco-2 cells (Figure 4). Meanwhile RAMEA had its IC50 value of 78.6 mM, the complex had no definable IC50 up to 100 mM. Based on this difference we presumed that α-CDs are really able to form inclusion complexes with phosphatidylcholine as one of the main membrane constituents. This complexation can drive to membrane injuries and an increase of membrane permeability, which can result in cell death.

### 2.2. Discussion

In our study, the toxic effect of α-CD derivatives was investigated *in vitro*, on Caco-2 cells and in parallel on human erythrocytes by two different methods: viability tests (MTT and hemolysis) and impedance-based cytotoxicity assay (RT-CES). The IC50 and HC50 values were determined and correlated with the structures. Mainly, the MTT assay is based on the enzymatic conversion of MTT in the mitochondria, and detects early cytotoxicity compared to the hemolysis and RT-CES [31]. Hemolysis studies were a simple and rapid investigation to classify the CDs according to their cytotoxicity [15]. Both MTT and hemolysis tests are suitable for the morphological verification of the toxic concentrations of α-CD, but could not provide kinetic data [32]. They are endpoint determinations, and higher concentrations of excipients may interfere with these detection methods [33]. Real-time cell electronic sensing assay supports the determination of toxic concentration values in a broad time interval and is a convenient method to characterize the kinetic factors of cytotoxicity [34]. Toxic concentrations of, sulfated and carboxymethylated derivatives have been determined by RT-CES, but these compounds were not toxic by MTT and hemolysis methods. These differences may be explained by the longer exposition time compared to the other assays. It has been confirmed that real-time assay set up over a broad time frame, from minutes to several hours, is the most accurate method for the determination of toxic concentrations of α-CDs.

Comparing the endpoint detection technique (MTT) and the non-invasive cell-based assay (RT-CES), the RT-CES method monitors cellular events in real-time without the incorporation of label or transformation of cells. The MTT test is advisable only for the preliminary phase of a cell death study. [32] Cell index shows the actual cell viability status and has been used in a number of cell morphology, adhesion, cell proliferation and receptor activity studies. The resulting data are biologically relevant because the elimination of labels brings the cells closer to physiological conditions. It has been confirmed by the study of Ozsvári *et al.* [34] who observed similar results of cytotoxicity using immortalized and rat primary cell lines in RT-CES experiments. The correlation of *in vitro* cytotoxicity values IC50 and *in vivo* LD50 was verified in the study of Boyd *et al.* [35]. The limitations of conventional cell-based assays was demonstrated, because dye-based endpoint detection only provides information about cell death (cell viability). Multiple parameters under dynamic circumstances are needed for predicting human acute toxicity studies [36]. Real-time cell electronic sensing assays provide reliable and dynamic cytotoxic parameters. The RT-CES method was the most sensitive technique in our study to determine the safest concentration of different α-CD derivatives. However, the cytotoxicity ranking obtained was the same as measured by MTT on the same cell line (Caco-2) and determined by the hemolysis test on human erythrocytes. Correlation was found among these tests based on different cell lines and different endpoints. The RT-CES method has been validated by Zhu *et al.* [37]. High cytotoxicity information content and more predictable kinetic data in human experiments were established.

Methylated (TRIMEA, RAMEA) and succinylated derivative (SuACD) showed the highest cytotoxicity. Substitution of succinyl group and of methyl groups resulted in higher toxicity in general, compared to the native α-CD. In the case of methylation, increased toxicity of TRIMEA related to RAMEA has been explained by the difference in the substitution degree (DS 18 and 11, respectively). Higher DS with the same alkyl substituents probably increased the toxicity of derivatives by reducing the free OH-groups. Ono *et al.* [38] investigated the cytotoxicity of maltosyl- α-, β-CDs compared to native α-, β- and γ-CDs on Caco-2 cells. Native α-CD possessed the highest cytotoxic attributes, but maltosyl groups suppressed the toxicity. The number and the position of methyl groups also determined the cytotoxicity in the case of β-CD: above a number of methyl substituents (DS ~ 10) the methylated β-CDs are highly toxic [11]. In our studies random methylation decreased, but full substitution dramatically increased the toxicity. On the contrary, the ionic substituents reduced the toxicity except phosphated α-CD. The viscosity of the solutions at appropriate concentrations may be the cause of the relatively high toxicity of the polymer derivative compared to its monomeric analog (CMACD). Hydroxypropyl and acetylated α-CDs are the most favorable derivatives in our investigations.

It is well known that hydroxypropyl groups reduce the toxic properties of native α-, β- and γ-CD [15,39]. However, the intensity of the cytotoxicity of CDs differs among the cell types, probably due to the difference in the membrane compositions of the cells [40]. The possible mechanism is that CDs can interact with plasma membranes by the extraction of different components into their cavity via inclusion complex formation [8]. Significant correlation was observed between the cytotoxicity determined by MTT on Caco-2 cells, hemolytic activity and the cholesterol complexation capacity of various β-CDs [26]. β-CDs can extract cholesterol from the lipid rafts and randomly methylated β-CDs can enter into intestinal epithelial cells by endocytosis [41]. The mechanism of α-CD cytotoxicity is different because α-CD cannot include the large cholesterol molecules. The acyl chain of phospholipids, however, fits into the tight hydrophobic cavity of smaller α-CD [13]. In our indirect test the randomly methylated α-CD-phosphatidylcholine complex did not result in toxicity on Caco-2 cell monolayer compared with the “empty” RAMEA, proving that there is no toxicity when the cavity of the CDs is no more available. However, phosphatidylcholine extraction alone does not explain for the possessed cytotoxicity of α-CDs. Phosphatidylserine, phosphatidylethanolamine, phosphatidylinositol and sphingomyelin also shows strong, but selective affinity for α-CDs [13].

Phospholipids can also be found in the erythrocyte external and internal bilayers in different ratios [13]. This explains that some of the investigated α-CD derivatives demonstrated concentration- dependent hemolysis. The highest hemolysis values were induced by methylated and succinylated derivatives in accordance with the MTT results. Hydroxypropyl and acetyl groups in the molecules diminished the toxicity (HC50 > 100 mM). The experiments of Motoyama *et al.* [42] confirmed our results, because HPACD was also less toxic on rabbits’ red blood cells than α-CD. All authors agree with the mechanism published by Irie *et al.* and Bost *et al.* [40,43]: inclusion complexation of the membrane components induces their release and the lysis of the erythrocytes or the irreversible damage of Caco-2 cells.

Comparing the HC50 with IC50, HC50 values are lower than IC50 values, which may indicate that α-CD derivatives interact more strongly with the erythrocyte membrane than with the membrane of intestinal cells. The degree of the cytotoxicity of CDs toward Caco-2 cells increased in the order of γ < β < α. [44]. In our previous study, lower IC50 values, that is higher toxic concentrations of different β-CD derivatives, were measured (up to 200 mM) than in the case of α-CDs in accordance with the above statement. Matilainen *et al.* [14,41] concluded that in terms of their toxicity changing in the order of γ < β < α, γ-CD was the safest to the Calu3 cells, too. Interestingly, the cytotoxicity of α-CD toward human corneal epithelial cells was even greater than that of dimethyl-β-CD (DIMEB), one of the most toxic methylated β-CD derivative [44]. These results suggest that the intensity of the cytotoxicity differs between the cell types due to the possibly different mechanism of membrane constituent extraction.

## 3. Experimental Section

### 3.1. Materials and Methods

α-Cyclodextrin derivatives were generously offered by Cyclolab Ltd. (Cyclodextrin Research & Development Laboratory, Budapest, Hungary). The derivatives used were: phosphated α-CD sodium salt, carboxymethylated α-CD sodium salt (CMACD), sulfated α-CD carboxymethylated α-CD polymer crosslinked with epichlorohydrin (CMACDEp), randomly methylated α-CD (RAMEA), acetylated α-CD (AcACD), hexakis-(2,3,6-tri-*O*-methyl)-α-CD (TRIMEA), (2-hydroxy) propyl α-CD (HPACD) and succinylated α-CD (SuACD). We investigated each α-CD in 7 different concentrations: 0.01; 0.1; 1; 10; 25; 50 and 100 mM, with the exception of TRIMEA, which was not soluble above 30 mM. The solid samples were dissolved in isotonic phosphate buffered saline (PBS), purchased from Sigma–Aldrich (Budapest, Hungary) (Table 2.)

**Table 2:** Chemical description of used α-cyclodextrin derivatives (DS = substitution degree).

α-Cyclodextrin Derivative	Short Name	Molecular Formula	Molecular Weight	DS
native		C_36_H_60_O_30_	972.84	0
random methyl	RAMEA	C_47_H_82_O_30_	1126.9	~11
hexakis(2,3-tri-*O*-methyl)	TRIMEA	C_54_H_96_O_30_	1225.4	18
(2-hydroxy)propyl	HPACD	C_49.5_H_87_O_34.5_	1234.3	~4.5
sulfated Na-salt	sulfated	C_36_H_48_O_66_S_12_Na_12_	2197.4	~12
phosphated Na-salt	phosphated	C_36_H_60_O_42_P_4_Na_4_	1380.7	~2–6
carboxymethylated Na-salt	CMACD	C_48_H_63_O_36_Na_3_	1212.9	~3.5
succinylated	SuACD	C_52_H_76_O_42_	1373.2	~4
acetylated	AcACD	C_52_H_76_O_38_	1267.1	~7
carboxymethyl-α-CD	CMACDEp	55 kDa		
crosslinked with epichlorohydrin				

### 3.2. Cell Culture

Caco-2 cell line was obtained from the European Collection of Cell Cultures (ECACC). Cells were grown in plastic cell culture flasks in Dulbecco’s Modified Eagle’s Medium supplemented with 3.7 g/L NaHCO_3_, 10% (*v*/*v*) heat-inactivated fetal bovine serum (FBS), 1% (*v*/*v*) non-essential amino acids solution, 1% (*v*/*v*) l-glutamine, 100 IU/mL penicillin, and 100 µg/mL streptomycin at 37 °C in an atmosphere of 5% CO_2_. The cells were routinely maintained by regular passaging. For cytotoxic and transport experiments, cells were used between passage numbers 20 and 40. The culture media were replaced with fresh media in every 72 h [45,46].

### 3.3. Hemolysis Test

Hemolysis test was performed on fresh human blood. Erythrocytes were separated from citrated blood by centrifugation at 2500× *g* for 10 min.; washed three times with PBS and resuspended in the same solution. Aliquots of the cell suspension with the respective red blood cell number of 5 × 10^7^ were added to the buffer solution (PBS pH 7.2) containing increasing concentrations of the samples investigated in the study. After mixing them gently, each solution was incubated at 37 °C for 10 min and then centrifuged at 5000× *g*. Finally, the absorbance of the hemoglobin released into the supernatant was measured at 540 nm with a FLUOstar OPTIMA Microplate Reader. The percentage of hemolysis was expressed as the ratio of hemoglobin in the supernatant of the sample solutions related to the hemoglobin concentration after the complete hemolysis of erythrocytes in water. The dose-response curve was determined, and the concentration inducing hemolysis in 50% of the erythrocyte population (HC50) was subsequently calculated [47].

### 3.4. MTT Cell Viability Assay

The MTT assay was performed on Caco-2 cells. The cells were seeded in 96 well plates until the cell monolayer become confluent. The medium was changed once. After one week the medium was removed, the cells were washed with PBS and exposed to increasing concentrations of α-CDs dissolved in phosphate buffer saline (PBS). Cells were incubated for 30 min on 37 °C. Control groups were processed equally and incubated without CD simultaneously. After treatment MTT dye (3-(4,5-dimethylthiazol-2-yl))-2,5-diphenyltetrazolium bromide, 5 mg/mL) was applied to each well for 3 h. MTT solution was removed and isopropanol-hydrochloric acid (25:1) was added to dissolve the formed formazan crystals. The absorbance was measured at 570 nm against a 690 nm reference with a FLUOstar OPTIMA Microplate Reader. Cell viability was expressed as the percentage of untreated control [48].

### 3.5. Real-Time Cell Microelectronic Sensing (RT-CES)

Real-time cell electronic sensing (RT-CES) is a label-free technique for dynamic monitoring of living cells [21]. The xCELLigence system (Roche, Basel, Switzerland) utilizes an electronic readout called impedance to non-invasively quantify adherence cell proliferation and viability. E-plates (Roche) contain gold microelectronic sensor arrays. The interaction between cells and the electrode generates impedance response that correlates linearly with cell index reflecting cell number, adherence and cell growth. This method is more sensitive and informative than colorimetric end-point assays to test pharmaceutical excipients [32,49].

The E-plate was coated with 0.2% rat tail collagen–DW solution for 20 min at 37 °C. Culture media (60 μL) was added to each well for background readings than 100 µL Caco-2 cell suspension was dispensed at the density of 1.5 × 10^4^ cells/well. The cells were grown for 2 days. The medium was changed to excipients solutions and the cells in the E-plate were kept in an incubator at 37 °C for 8 h and monitored every 5 min.

The cell index at each time point was defined as (Rn − Rb)/15, where Rn is the cell-electrode impedance of the well when it contains cells and Rb is the background impedance of the well with the media alone.

### 3.6. Statistical Analysis

All data were obtained from three to five independent biological replicates and in the same experiments, four parallel concentrations (wells) were measured. Raw data of cell viability (difference of absorbance values at 570 nm against 690 nm), hemolysis (absorbance at 540 nm), and cell index were compared with one-way ANOVA (using Geisser-Greenhouse correction) followed by Tukey post-testing (because all data passed the D’Agostino and Pearson omnibus normality test). For this purpose, data related to five concentrations were selected. Statistical significance for the difference of means was assigned into one of five categories: *p* > 0.05 (not significant), *p* < 0.05 (*), *p* < 0.01 (**), *p* < 0.001 (***) or *p* < 0.0001 (****). Data presented in this paper are expressed as mean ± SD. Statistical analysis was performed with GraphPad Prism 6.05, while other calculations were made by means of Microsoft Office Excel 2013. The results of statistical analysis in the case of MTT and hemolysis tests are shown in the Table 3 and Table 4. The results of statistical analysis of RT-CES is presented in Figure 3.

**Table 3:** Comparison of cell viability values among the groups treated with different α-CD derivatives (the number of asterisks indicates the level of statistical significance, *p* < 0.005 (*), *p* < 0.001 (**), *p* < 0.001 (***), *p* < 0.0001 (****).

Logarithm of Concentrations	−4	−3	−2	−1602	−1301
HPACD *vs.* Phosphated	**				
HPACD *vs.* Polymer	*				
HPACD *vs.* SuACD			*		
Sulphated *vs.* Phosphated	**			****	****
Sulphated *vs.* SuACD				****	****
Phosphated *vs.* AcACD	**				
Phosphated *vs.* CMACD			**	***	****
Phosphated *vs.* SuACD			***		
Phosphated *vs.* Polymer			*	**	****
AcACD *vs.* Polymer	*				
RAMEA *vs.* TRIMEA		*	****	****	
RAMEA *vs.* HPACD		*	***	***	**
RAMEA *vs.* Sulphated		*	***	**	
RAMEA *vs.* AcACD		**	****	****	**
RAMEA *vs.* Phosphatidylcholine + RAMEA		**	****	***	**
RAMEA *vs.* Phosphated			****	****	****
RAMEA *vs.* SuACD				****	****
RAMEA *vs.* Polymer				*	
native *vs.* RAMEA			**	***	****
native *vs.* TRIMEA			****	****	
native *vs.* Phosphated				*	
native *vs.* SuACD				***	
native *vs.* Sulphated					**
native *vs.* CMACD					**
native *vs.* Polymer					*
TRIMEA *vs.* Sulphated			****	****	
TRIMEA *vs.* Phosphated			***		
TRIMEA *vs.* CMACD			****	****	
TRIMEA *vs.* SuACD			****		
TRIMEA *vs.* Polymer			****	****	
SuACD *vs.* AcACD			*		
SuACD *vs.* Polymer				****	****
CMACD *vs.* SuACD				****	****

**Table 4:** Comparison of hemolysis values among the groups treated with different α-CD derivatives (the number of asterisks indicates the level of statistical significance. *p* < 0.005 (*), *p* < 0.001 (**), *p* < 0.001 (***), *p* < 0.0001 (****).

Logarithm of Concentration	−2	−1602	−1301	−1
native *vs.* RAMEA	*		**	**
native *vs.* TRIMEA	****		****	****
native *vs.* HPACD	****	****	****	****
native *vs.* Sulfated	**	****	****	****
native *vs.* Phosphated		****	****	****
native *vs.* CMACD		****	****	****
native *vs.* SuACD	****	****	****	****
native *vs.* AcACD	**	****	****	****
native *vs.* CMACDEp	***	****	****	****
RAMEA *vs.* TRIMEA	****	*	****	****
RAMEA *vs.* HPACD	****	****	****	****
RAMEA *vs.* Sulfated	****	****	****	****
RAMEA *vs.* Phosphated		****	****	****
RAMEA *vs.* CMACD		****	****	****
RAMEA *vs.* SuACD	****	****	****	****
RAMEA *vs.* AcACD	****	****	****	****
RAMEA *vs.* CMACDEp	****	****	****	****
TRIMEA *vs.* HPACD	****	****	****	****
TRIMEA *vs.* Sulfated	****	****	****	****
TRIMEA *vs.* Phosphated	****	****	****	****
TRIMEA *vs.* CMACD	****	****	****	****
TRIMEA *vs.* SuACD	****	****	****	****
TRIMEA *vs.* AcACD	****	****	****	****
TRIMEA *vs.* CMACDEp	****	****	****	**
HPACD *vs.* Phosphated	****			
HPACD *vs.* CMACD	****			
HPACD *vs.* SuACD	****	****	****	***
HPACD *vs.* AcACD				*
HPACD *vs.* CMACDEp		****	****	****
Sulfated *vs.* Phosphated	**			
Sulfated *vs.* CMACD	***			
Sulfated *vs.* SuACD	****	****	****	*
Sulfated *vs.* CMACDEp		****	****	****
Phosphated *vs.* SuACD	****	****	****	
Phosphated *vs.* AcACD	**			
Phosphated *vs.* CMACDEp	****	****	****	****
CMACD *vs.* SuACD	****	****	****	
CMACD *vs.* AcACD	**			
CMACD *vs.* CMACDEp	****	****	****	****
SuACD *vs.* AcACD	****	****	****	
SuACD *vs.* CMACDEp	****		****	****
AcACD *vs.* CMACDEp		****	****	****

## 4. Conclusions

It has been concluded that synthetic modifications on the glucopyranose rings of α-CD may enhance or reduce its cytotoxic effects. Methyl and succinyl substitution may increase the toxicity depending on the number of substituents, but hydroxypropyl α-CDs like β-CDs are suitable, even for parenteral formulations and also acetylation definitely reduces the toxic effect. Real-time kinetic assays provided quantitative, time dependent and more specific cytotoxicity values than the conventional MTT viability and hemolysis tests. Our results indicate that some α-CDs could be safe in pharmaceutical formulations. The same tests will be done on new generation of α-CD derivatives with a well-defined substitution degree, in order to complete this structure-activity study.
